# Exosomal linc-FAM138B from cancer cells alleviates hepatocellular carcinoma progression via regulating miR-765

**DOI:** 10.18632/aging.202430

**Published:** 2020-12-26

**Authors:** Chenyi Zhuo, Tingzhuang Yi, Jian Pu, Xiaoning Cen, Yang Zhou, Shi Feng, Cheng Wei, Pengyu Chen, Wei Wang, Chongchan Bao, Jianchu Wang, Qianli Tang

**Affiliations:** 1 General Surgery, Affiliated Hospital of YouJiang Medical University For Nationalities, Baise 533000, Guangxi, China; 2 Gastrointestinal Medicine, Affiliated Hospital of YouJiang Medical University For Nationalities, Baise 533000, Guangxi, China; 3 YouJiang Medical University For Nationalities, Baise 533000, Guangxi, China

**Keywords:** hepatocellular carcinoma, exosome, linc-FAM138B, miR-765, tumorigenesis

## Abstract

Exosomes are small vesicles with a diameter of 30-150 nm secreted by cells, which can be used as signal carriers to transfer nucleic acids, proteins, lipids and other functional substances to the recipient cells and play a role in cell communication. Hepatocellular carcinoma is the fourth most common cause of cancer-related death worldwide. Studies have shown that long non-coding RNAs (lncRNAs) are involved in the development and progression of many types of tumors. Our present study found that linc-FAM138B was reduced in HCC tissues and cell lines, low expression of linc-FAM138B indicated a poor prognosis in HCC patients. Interestingly, linc-FAM138B could be packaged into cancer cells. And exo-FAM138B inhibited the proliferation, migration and invasion of HCC cells. Furthermore, linc-FAM138B sponged miR-765 levels. And exo-si-FAM138B promoted HCC progression, while deletion of miR-765 reversed the role of exo-si-FAM138B. In vivo tumorigenesis experiments showed that exo-FAM138B suppressed HCC growth via modulating miR-765. In conclusion, exo-linc-FAM138B secreted by cancer cells inhibited HCC development via targeting miR-765, which provided a new idea and perspective for in-depth understanding of the complex signal regulation in HCC process.

## INTRODUCTION

About 75-85% of liver cancer in the world is hepatocellular carcinoma (HCC) [1]. HCC is the sixth most common cancer in the world and the fourth most common cause of cancer-related death worldwide. The incidence and mortality of malignant tumors of HCC in China are the fourth and the third, respectively [2]. HCC has a poor prognosis and a high risk of recurrence [3]. At present, the diagnostic methods of HCC are limited, and it is easy to miss early small tumors. However, the traditional treatment of HCC is not sufficient [4–6]. Therefore, patients with HCC need effective methods for diagnosis, treatment and monitoring of tumor progression.

Exosomes are membranous extracellular vesicles with a diameter of 30-100 nm released by living cells, which exist in various body fluids such as blood, urine and saliva [7]. Exosomes contain bioactive molecules such as nucleic acids, proteins and lipids [8]. The exosome transmits the bioactive components to the receptor cells, regulates the receptor cells' behavior, and exerts its biological function [9]. The tumor microenvironment consists of tumor cells, fibroblasts, immune cells, endothelial cells and other cellular components, as well as extracellular matrix, growth factors, inflammatory cytokines, proteolytic enzymes and their inhibitors. The malignant behavior of tumor cells depends not only on the tumor cells themselves, but also on the communication between tumor cells and the tumor microenvironment [10]. More and more studies have found that ncRNAs from exosomes promote the occurrence and development of HCC [11].

LncRNAs are non-coding RNAs with a length of more than 200bp [12]. It was confirmed that lncRNA exerts an essential role in the growth and metastasis of HCC [13]. LncRNA plays its regulatory role mainly through the following ways: first, it interacts with protein directly; second, it interacts with miRNA as ceRNA; third, it encodes proteins or peptides; and fourth, it participates in intercellular communication through exosome. Takahashi et al. [14] showed that lncRNA-VLDLR can participate in cell-to-cell communication through exosomes derived from tumor cells and regulate the drug resistance of recipient liver cancer cells to chemotherapy. Exosomal lncRNA H19 promoted the activation of hepatic stellate cells and cholestatic liver fibrosis, which may eventually lead to end-stage liver diseases, such as liver cirrhosis and liver malignant tumors [15]. LncRNA-ATB, also known as lncRNA activated by TGF- β, is the first lncRNA, found to be activated by transforming growth factor (TGF). LncRNA-ATB is highly expressed in hepatocellular carcinoma, colon cancer, prostate cancer and lung cancer, but down-regulated in pancreatic cancer [16]. These studies demonstrated that lncRNA-ATB might become an important marker for tumor diagnosis, treatment and prognosis. Lee et al. [17] proved that exosomal lncRNA-ATB is a new prognostic marker and therapeutic target for HCC. Conigliaro et al. [18] found that CD90+ hepatoma cells regulate the phenotype of vascular endothelial cells and affect their tumor microenvironment by releasing exosomes containing lncRNA H19. Exosomal lncRNA H19 may be a new therapeutic target to reduce the recurrence and metastasis of hepatocellular carcinoma.

LncRNA FAM138B (linc-FAM138B) was first found to be a diagnostic and prognostic biomarker in lung adenocarcinoma [19]. A recent study indicated that linc-FAM138B was related to the development and prognosis of HBV-related hepatocellular carcinoma [20]. However, the function of linc-FAM138B in HCC is poorly identified. Present study showed that linc-FAM138B was down-regulated in HCC tissues, and linc-FAM138B could be enveloped into exosomes from cancer cells. Furthermore, exo-FAM138B suppressed the growth and invasion of HCC cells through targeting miR-765.

## RESULTS

### Linc-FAM138B is reduced in HCC tissues

**Figure 1:**
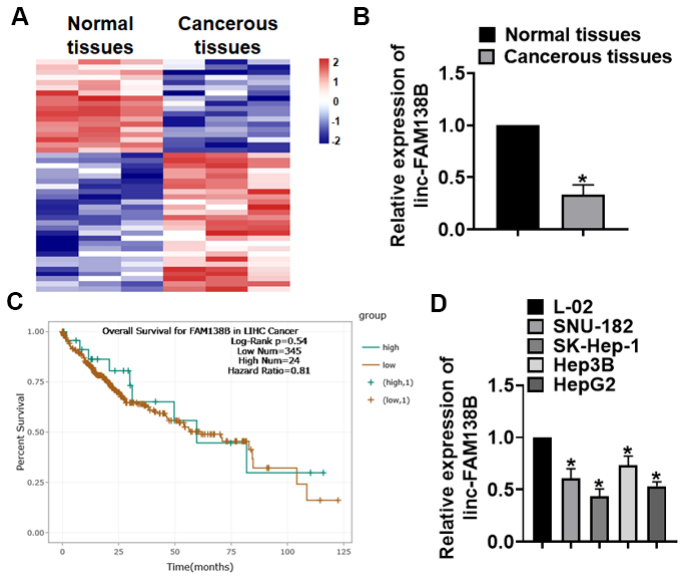
**The expression of linc-FAM138B in HCC tissues and cells.** (**A**) LncRNA expression profiles in normal and cancer tissues of HCC. (**B**) We collected 40 samples of patients diagnosed with HCC. The expression of linc-FAM138B in normal and cancerous tissues was detected by qRT-PCR. n = 40. (**C**) The overall survival rate of HCC patients with high or low linc-FAM138B expression was download from ENCORI database. (**D**) qRT-PCR analysis for linc-FAM138B level in HCC cell lines (Hep3B, HepG2, SNU-182 and SK-Hep-1)) and normal hepatocytes (L-02). Data are mean ± SD; *P < 0.05. Data among multiple groups were analyzed by one-way ANOVA, followed by a Tukey post hoc test. The experiment was repeated in triplicate.

HCC patients’ tissues were collected, and we performed microarray to identify the differential expressed lncRNAs, which showed a decrease of linc-FAM138B in HCC tissues (Figure 1A). Then, qRT-PCR analysis confirmed the low expression of linc-FAM138B HCC tissues (Figure 1B). The survival rate of patients with HCC directly reflects the level of prognosis, and we used ENCORI database to analyze the survival rate of HCC patients with high and low expression of linc-FAM138B. And the data indicated that low expression of linc-FAM138B patients had a poorer prognostic level (Figure 1C). We also examined linc-FAM138B level in different HCC cell lines, which showed that linc-FAM138B was decreased in HCC cell lines (Hep3B, HepG2, SNU-182 and SK-Hep-1) comparing with normal human hepatocyte cells (L-02) (Figure 1D).

### Linc-FAM138B was packaged into exosomes and derived from cancer cells

**Figure 2:**
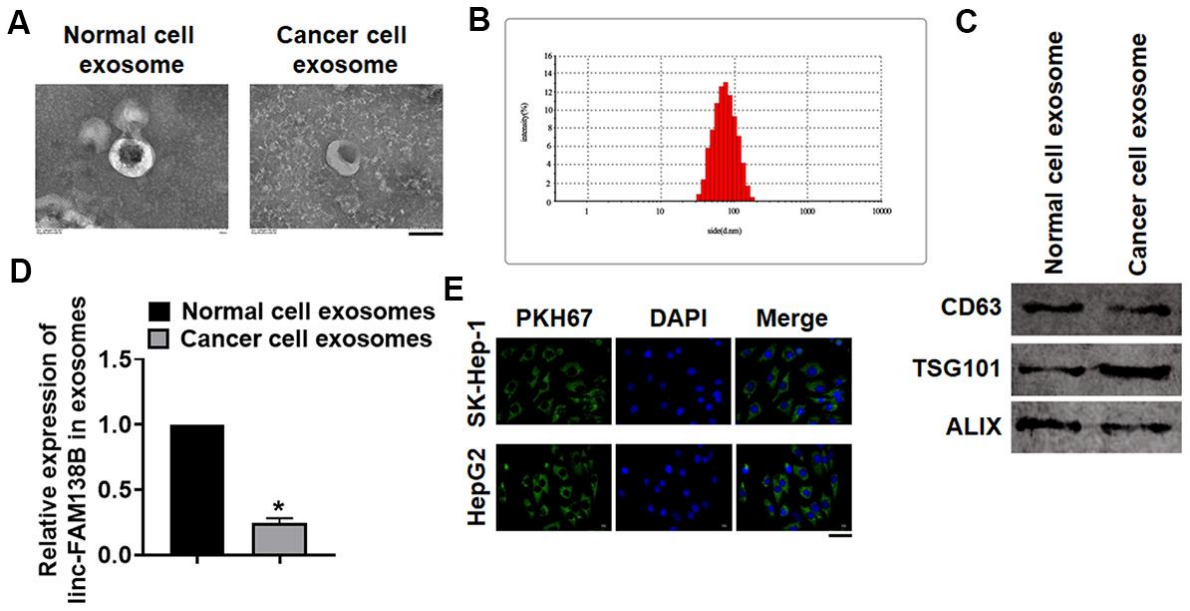
**Linc-FAM138B was packaged into exosomes from cancer cells in HCC.** Normal and cancer cells were isolated from HCC tissues, then exosomes were isolated from the supernatant of normal and cancer cells. (**A**) TEM images of exosomes isolated from normal and cancer cells. Scale bar, 100 nm. (**B**) Zetasizer Nano ZS was used to detect the diameter of isolated exosomes. (**C**) Western blot for exosome markers CD63, Tsg101 and Alix. (**D**) The expression of linc-FAM138B in exosomes from normal cells and cancer cells was tested by qRT-PCR. (**E**) PKH67 labeled linc-FAM138 was transfected into tumor cells. Then, SK-Hep-1 and HepG2 were incubated with exosomes from supernatant of tumor cells. And immunofluorescence experiment indicated a dominant fluorescence intensity of PKH67 in SK-Hep-1 and HepG2 cells. Scale bar, 100 μm. Data are mean ± SD; *P < 0.05. Data among multiple groups were analyzed by one-way ANOVA, followed by a Tukey post hoc test. The experiment was repeated in triplicate.

To identify the origin of linc-FAM138B in HCC, we isolated cells from normal and cancerous tissues of HCC. Then, exosomes were isolated from isolated cells, and TEM was used to detect exosomes morphology and structure (Figure 2A). And Zetasizer Nano ZS exhibited that the diameter of isolated exosomes was approximately 70 nm (Figure 2B). As well, western blot tested exosome marker genes CD63, TSG101 and ALIX (Figure 2C). These data indicated that we successfully extracted exosomes. Then, we detected the linc-FAM138B level in exosomes from normal and cancer cells, which showed that linc-FAM138B was lower expressed in cancer cells of HCC (Figure 2D). Moreover, we transfected PKH67 labeled linc-FAM138 into tumor cells. Then, SK-Hep-1 and HepG2 were incubated with exosomes from supernatant of tumor cells. And immunofluorescence experiment indicated a dominant fluorescence intensity of PKH67 in SK-Hep-1 and HepG2 cells (Figure 2E). Together, linc-FAM138B could be enveloped into exosomes of cancer cells in HCC patients, and exosome from cancer cell transmitted linc-FAM138B to HCC cell lines.

### Exosomal-linc-FAM138B inhibited growth of cancerous hepatocytes

**Figure 3:**
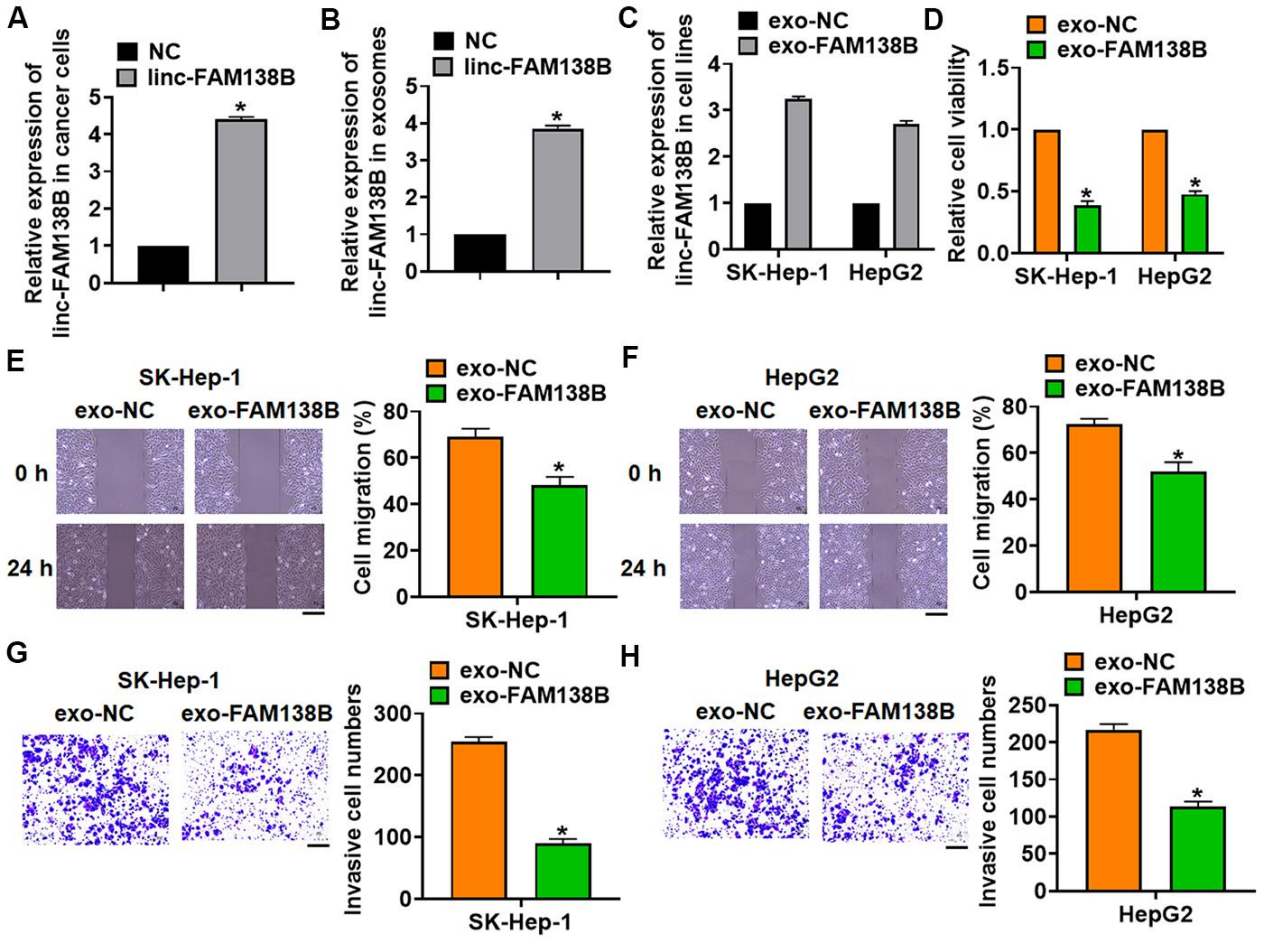
**Exosomal linc-FAM138B inhibited the proliferation, migration and invasion of HCC cells. Cancer cells were isolated from HCC tissues.** (**A**) Cancer cells were transfected with linc-FAM138B or NC, qRT-PCR was to determine transfection efficiency. (**B**) Exosomes were isolated from cancer cells after transfection, qRT-PCR was to determine linc-FAM138B expression in isolated exosomes. (**C**) SK-HEP-1 and HepG2 cells were incubated with isolated exosomes, and the expression of linc-FAM138B in SK-HEP-1 and HepG2 cells was detected using qRT-PCR. (**D**) MTT assay was to detected proliferation of SK-HEP-1 and HepG2 cells. (**E**, **F**) Wound healing assay was to evaluate migration of SK-HEP-1 and HepG2 cells. Scale bar, 60 μm. (**G**, **H**). Transwell assay was to examine invasion of SK-HEP-1 and HepG2 cells. Scale bar, 60 μm. Data are mean ± SD; *P < 0.05. Data among multiple groups were analyzed by one-way ANOVA, followed by a Tukey post hoc test. The experiment was repeated in triplicate.

To evaluate the role of exosomal-linc-FAM138B (exo-FAM138B) in HCC development, we transfected linc-FAM138B plasmid or its NC into cancer cells (Figure 3A and Supplementary Figure 1). And we isolated exosomes from cancer cells after transfection, the expression of linc-FAM138B was upregulated in exosomes after linc-FAM138B plasmid transfection (Figure 3B). Then, cancerous cell lines (SK-Hep-1 and HepG2) were incubated with exosomes from cancer cells transfected linc-FAM138B plasmid or its NC. And linc-FAM138B was increased in SK-HEP-1 and HepG2 cells after incubation (Figure 3C), which indicated that inc-FAM138B could be transmitted by exosomes. Functionally, MTT assay showed that exo-FAM138B reduced cell viability of SK-HEP-1 and HepG2 cells (Figure 3D). Furthermore, wound healing assay suggested that exo-FAM138B inhibited cell migration in SK-HEP-1 and HepG2 cells (Figure 3E, 3F). Transwell assay showed that exo-FAM138B suppressed cell invasion in SK-HEP-1 and HepG2 cells (Figure 3G, 3H). Together, exo-FAM138B secreted by MSCs promoted tumor progression in TNBC cells.

### Linc-FAM138B competitively inhibited miR-765 expression

**Figure 4:**
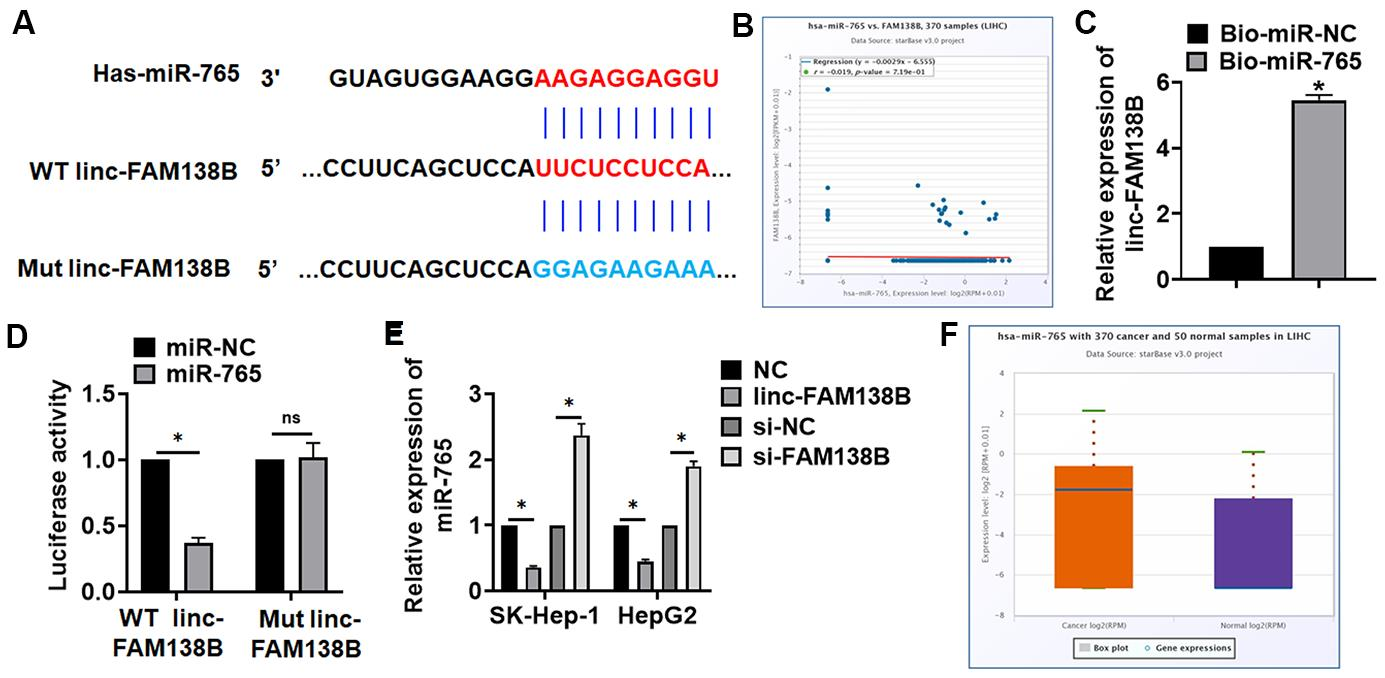
**Linc-FAM138B acted as a sponge of miR-765.** (**A**) MiRanda database showing the binding sites of miR-765 with linc-FAM138B, and the mutant sequence of linc-FAM138B. (**B**) StarBase showed a negative correlation between linc-FAM138B and miR-765 in HCC. (**C**) Biotinylated miR-765 or NC was transfected into HepG2 cells, and qRT-PCR was performed to detect the enrichment of linc-FAM138B. (**D**) Wild type and mutant linc-FAM138B was transfected into HEK293 cells with miR-765 or miR-NC, and luciferase assay was to evaluate the binding between miR-765 and linc-FAM138B. (**E**) SK-HEP-1 and HepG2 cells were transfected with linc-FAM138B plasmid or si-linc-FAM138B or its NC, the mRNA level of miR-765 was detected using qRT-PCR. (**F**) The expression of miR-765 was identified in HCC patients using database. Data are mean ± SD; *P < 0.05, #P < 0.05 vs cancer exo. Data among multiple groups were analyzed by one-way ANOVA, followed by a Tukey post hoc test. The experiment was repeated in triplicate.

To clarify the mechanism, we used MiRanda database, and found a potential binding between linc-FAM138B and miR-765 (Figure 4A). And StarBase showed a negative correlation between linc-FAM138B and miR-765 in HCC (Figure 4B). Further, endogenous linc-FAM138B was enriched in biotinylated miR-765 transfected HepG2 cells, which reveals a direct binding of linc-FAM138B with miR-765 (Figure 4C). Then luciferase assay showed miR-765 inhibited activity of WT linc-FAM138B not Mut linc-FAM138B in HEK293 cells (Figure 4D). And overexpression of linc-FAM138B inhibited miR-765 level, while silencing of linc-FAM138B promoted miR-765 level (Figure 4E). Moreover, miR-765 was increased in HCC patients using database (Figure 4F).

### Silencing of linc-FAM138B promoted the growth of cancerous hepatocytes by inhibiting miR-765

**Figure 5:**
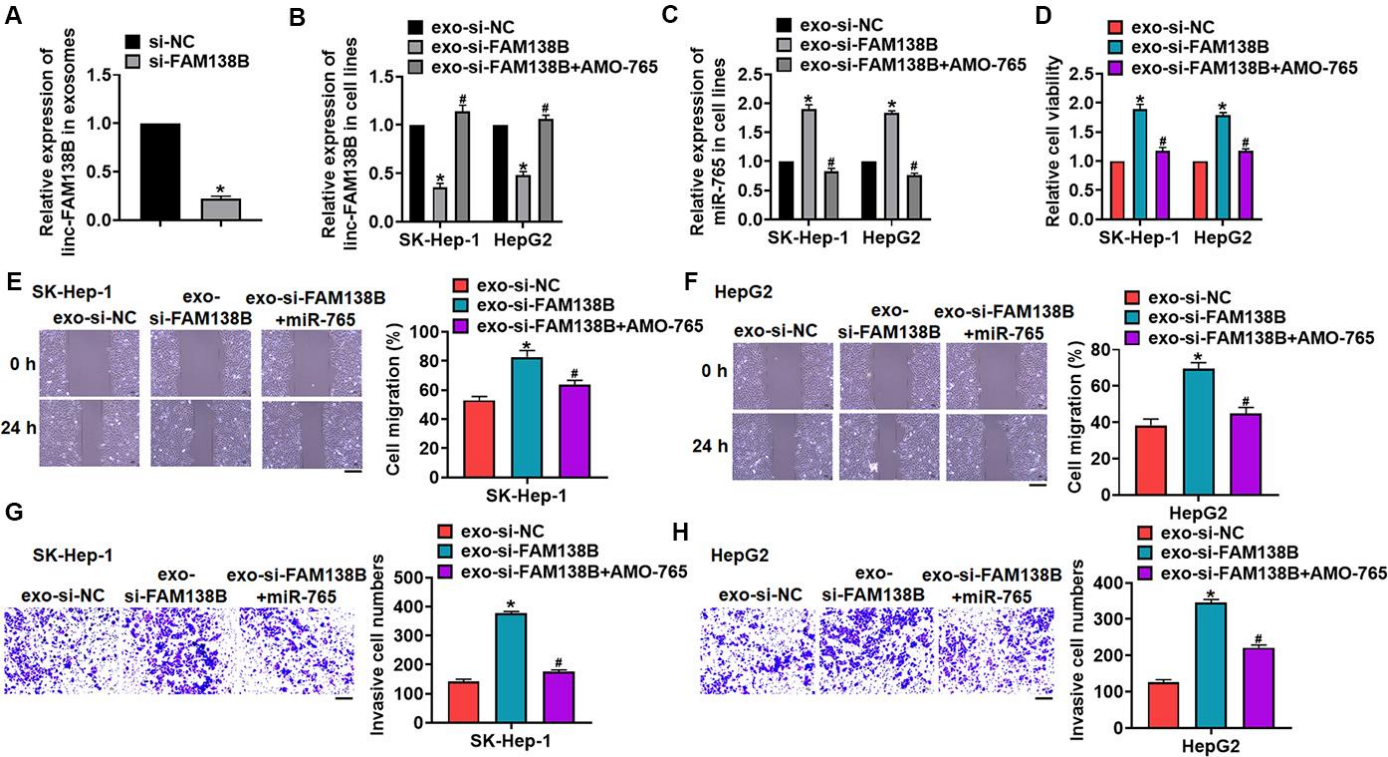
**Exo-si-FAM138B promoted HCC growth by promoting miR-765.** (**A**) Cancer cells was transfected with si-FAM138B or NC, and exosomes were isolated. The expression of linc-FAM138B in exosomes was determined. SK-HEP-1 and HepG2 cells were incubated with isolated exosomes, and then were transfected with AMO-765 or AMO-NC. (**B**) The expression of linc-FAM138B in SK-HEP-1 and HepG2 cells was evaluated. (**C**) The level of miR-765 in SK-HEP-1 and HepG2 cells was detected. (**D**). MTT assay for cell proliferation of SK-HEP-1 and HepG2 cells. (**E**, **F**). Wound healing assay for cell migration of SK-HEP-1 and HepG2 cells. Scale bar, 60 μm. (**G**, **H**). Transwell assay for cell invasion of SK-HEP-1 and HepG2 cells. Scale bar, 60 μm. Data are mean ± SD; *P < 0.05 vs exo-linc-FAM138B+NC. Data among multiple groups were analyzed by one-way ANOVA, followed by a Tukey post hoc test. The experiment was repeated in triplicate.

We then transfected siRNA of linc-FAM138B (si-FAM138B) into cancer cells from HCC tissues, and exosomes was isolated from cancer cells after transfection. Si-FAM138B transfection inhibited linc-FAM138B in exosomes (Figure 5A). Then, SK-HEP-1 and HepG2 cells was incubated with isolated exosomes, and exosomes containing si-FAM138B (exo-si-FAM138B) inhibited FAM138B expression in HCC cells, while promoted miR-765 expression (Figure 5B, 5C). And HCC cells were transfected with AMO-765 or AMO-NC with the presence of exo-si-FAM138B, AMO-765 reversed the effects of exo-si-FAM138B (Figure 5B, 5C). Followed functional analysis showed that exo-si-FAM138B promoted the cell proliferation, migration, invasion (Figure 5D–5H), while AMO-765 showed the opposite function (Figure 5D–5H).

### Exo-FAM138B inhibited HCC tumorigenesis by downregulating miR-765

**Figure 6:**
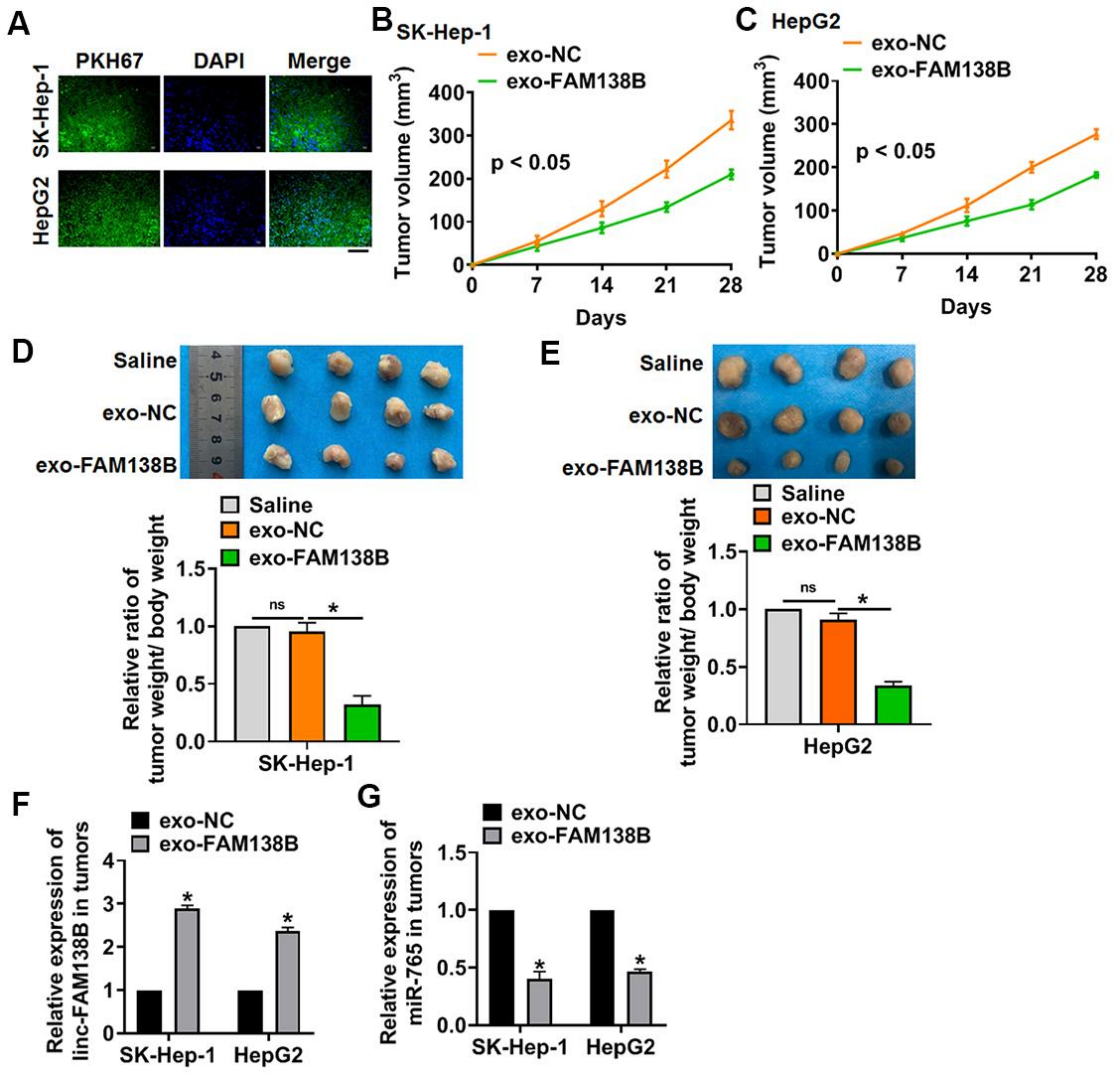
**Exo-FAM138B suppressed HCC growth in vivo. PKH67 labeled linc-FAM138 was transfected into tumor cells, then exosomes from tumor cells were isolated.** SK-HEP-1 and HepG2 cells were subcutaneously injected into nude mice. And a dosage of 5 mg exosomes or 10 μl saline was administered into mice via tail vein injection once every 3 days for 2 weeks. (**A**) Immunofluorescence assay showed a prominent fluorescence intensity of PKH67 in isolated tumor tissues. Scale bar, 100 μm. (**B**, **C**) Tumor volume was measured every 7 days. (**D**, **E**) Tumors was isolated after 28 days of SK-HEP-1 and HepG2 cells injection, and photos for representative tumors. The ratio of tumor wight to body weight of mice was calculated. (**F**) The level of linc-FAM138B in isolated tumors were detected by qRT-PCR. (**G**) The mRNA level of miR-765 in isolated tumors was tested. Data are mean ± SD; *P < 0.05. Data among multiple groups were analyzed by one-way ANOVA, followed by a Tukey post hoc test. The experiment was repeated in triplicate.

We implemented tumor formation in nude mice. HCC cells were subcutaneously injected into nude mice, then exo-FAM138B injected through tail vein. Exo-FAM138B injection decreased tumor volume (Figure 6A, 6B), and inhibited the ratio of tumor weight to body weight (Figure 6C, 6D). In addition, isolated tumor tissues had a higher linc-FAM138B level after exo-FAM138B injection (Figure 6E). Moreover, injection of exo-FAM138B reduced the mRNA level of miR-765 (Figure 6F). Thus, exo-FAM138B inhibited HCC growth by modulating miR-765 (Figure 6G).

## DISCUSSION

Primary HCC is the most common primary liver tumor, which has a very high incidence and mortality in China [21]. Clinical statistics show that the five-year survival rate of patients with liver cancer is not ideal. Although the development of antineoplastic drugs has improved the therapeutic effect of liver cancer, more than 600000 people still die of HCC each year [22]. Therefore, it is necessary to explore the pathogenesis of HCC and develop effective treatments. At present, researchers have found that many kinds of lncRNAs are near related to tumor growth, metastasis and recurrence [23]. For example, lncRNA-MEG3 shows abnormal expression in HCC tissues, and participates in the occurrence and development of HCC [24]. In present study, we collected tissue samples from HCC patients, and the lncRNA expression profiles showed a decrease of linc-FAM138B in HCC tissues. The abnormal expression of genes in the pathological state indicates its potential function as a prognostic tumor marker for patients [24]. Thus, we used ENCORI database, and found that patients with lower linc-FAM138B level had a poor prognosis. These data suggested linc-FAM138B might contribute to the progression of HCC, which prompted us to further explore linc-FAM138B function in HCC.

Recent studies have shown that exosomes can act as transport vesicles of functional lncRNA [25]. Because proteins and RNA are easily degraded outside the cell, exosomes can protect biological compounds from degradation outside the cell [26]. It has been proved that exosomes protect cells from radiation damage by ingesting survival proteins, thus promote cell proliferation and improve metastatic potential [27]. This discovery is of great significance for the clinical treatment of cancer. Besides, lncRNA TUC339 was identified in the exosome of HCC and showed high expression in the exosome [28]. Inhibition of this TUC339 in cells by RNA interference results in reduced cell proliferation, clone growth and cell adhesion. This observation shows that cells use exosomes and TUC339 to promote the proliferation of nearby cells. LncRNA ROR is highly expressed in exosomes of HCC cells treated with doxorubicin [29]. Exosomal lncRNA ROR increased the chemical resistance of HCC cells, which indicates that cancer cells may use lncRNA and exosomes to improve chemical resistance in neighboring cells. Interestingly, present data found that linc-FAM138B was packaged into cancer cells of HCC, and exo-FAM138B could be transmitted in to HCC cell lines. Functionally, exo-FAM138B suppressed the growth and invasion of HCC cells.

Moreover, our data showed that linc-FAM138B was a sponge of miR-765, and there was a binding between linc-FAM138B and miR-765. Exo-FAM138B inhibited HCC progression via modulating miR-765. MiR-765 is a highly conserved miRNA, and researches have shown the function of miR-765 in multiple cancer. For example, miR-765 inhibited the development of miR-765 by targeting PLP2 [30]. In addition, lncRNAs exert function in tumors mainly via modulating miRNAs expression. LncRNA PVT1 promoted gemcitabine resistance of pancreatic cancer via sponging miR-619-5p [31]. In vivo tumorigenesis experiments showed that exo-FAM138B inhibited HCC development via miR-765.

At present, exosome is used as a targeted drug delivery method, and clinical trials are being carried out. Most studies about exosomes focus on miRNAs and lncRNAs, which indicates a fascinating new field for tumor treatment.

## CONCLUSIONS

Our study revealed that exo-linc-FAM138B secreted by cancer cells inhibited HCC development via targeting miR-765, which provided a new idea and perspective for in-depth understanding of the complex signal regulation in HCC process.

## MATERIALS AND METHODS

### Tissue specimen

The surgical specimens of 40 HCC patients were collected, which were used for follow-up experimental detection. The experiment was permitted by the Ethics Review Committee of Affiliated Hospital of YouJiang Medical University For Nationalities and the patients signed informed consent.

### Animals

Animal experiments were permitted by the Animal Protection and Ethics Committee of YouJiang Medical University For Nationalities. BALB/c nude mice (6-8 weeks) were purchased from Beijing Weitong Lihua Experimental Animal Technology Co., Ltd. For the experiment of Xenograft, SK-Hep-1 and HepG2 cells (5 × 10^6^) were suspended in 200 μl normal saline and subcutaneously injected into right flanks. And a dosage of 5 mg (20ul) exosomes was administered into mice via tail vein injection once every 3 days for 2 weeks. Tumor volume (mm^3^): V (Mm^3^) = S2 (Mm^2^) × L (Mm) / 2.

### Exosome isolation and identification

Cells were isolated from cancer and adjacent normal tissues of HCC patients as previous described [32]. Briefly, each HCC tissue specimen was minced into 1 mm3 cube chunks and enzymatically dissociated to single cells. Exosomes were isolated from the culture medium by gradient centrifugation, as reported previously [33]. Following initial centrifugation for 30 min at 3000×g, cells and other debris were removed and the supernatant was harvested and centrifuged at 10,000×g for 30 min to remove microvesicles larger than exosomes. The supernatant was finally centrifuged at 110,000×g for 70min. The isolation process was performed at 4° C, and the exosomes were resuspended in PBS and stored at −80° C. Transmission electron microscopy (TEM) was used to identify exosomes structures. Exosomes were analyzed using exosome marker protein CD63, ALIX and TSG101 via Western blot.

### Cell culture and transfection

Cell lines were purchased from CHI Scientific, Inc (Jiangsu, China). The cells were cultured with complete medium including 89% 1640, 10% FBS and 1% Penicillin and streptomycin, both were purchased from Biological Industries (Beit-Haemek, Israel), and maintained in incubator with 37° C and 5% of CO_2_ saturated humidity. The cells were plated until the cell density reached 80% confluency of dishes to transfect. Plasmid of LINC-FAM138B or miR-765 inhibitor (AMO-765) or small interfering RNA (si-RNA) of linc-FAM138B were constructed by Genechem (Shanghai, China). The plasmids or siRNAs transfected with Lipofectamine 2000 (Invitrogen, Carlsbad, CA).

### qRT-PCR

RNA extraction was performed using trizol reagent. NanoDrop 8000 (Thermo Scientific, Waltham, MA, USA) was used to detect the concentration and purity of RNA. The single-stranded cDNAs were synthesized from 1 μg of RNA. The expression of mRNAs and miRNAs were quantified by RT-PCR with SYBR Green I (Thermo Fisher Scientific, Inc). GADPH used as internal control for normalizing linc-FAM138B expression, and U6 used as internal control for normalizing miR-765.

### Western blot

After RIPA cleavage, we extracted total protein and measured with BCA method. After quantitative denaturation, 60 μg proteins were loaded via SDS-PAGE (with a constant voltage of 110 V) and transferred onto nitrocellulose membranes (with a constant current of 300 mA), then the membrane containing proteins was blocked with 5% BSA. The first incubation and second incubation were carried out according to the operation steps. The expression of the protein was expressed by the gray value. Primary antibodies list: CD63 (ab134045, Abcam), Tsg101 (ab125011, Abcam), Alix (ab88388, Abcam).

### MTT assay

Cells were plated in 96-well plates and we used MTT assay to detect the cell viability. MTT (20 nmol/L; Beyotime Biotechnology, China) was added after 12 h of exosomes treatment, and incubated at 37° C for 4h. We measured the absorbance of 450 nm with 150 μL DMSO.

### Cell migration assay

The HCC cells were spread in a 6-well plate, and when they grew to 80%, horizontal lines were drawn in the cells with a ruler at an interval of 0.5cm, with 4 lines drawn in each well. The cells were washed with PBS for 3 times. Serum free culture medium was added and photographed at 0 hours. The cells were continuously cultured for 24 hours before the photo was taken.

### Cell invasion assay

First, the Matrigel was spread over the Transwell, and the starved HCC cells for 12 hours were inoculated into the upper chamber. The culture medium with serum was added to the lower chamber and cultured for 12 hours. The Transwell was taken out, the culture medium was discarded. And Transwell was washed using PBS, and fixed with methanol for 30 minutes. After the chamber was dried, the cells were stained with crystal violet for 20min, and the upper cells were removed and washed with PBS for 3 times. The cells were photographed and counted under the microscope.

### Luciferase assay

PsiCHECK-2 luciferase reporter plasmid was inserted with the wildtype Linc-FAM138B (WT-Linc-FAM138B) and mutant Linc-FAM138B (Mut-Linc-FAM138B) 3’UTR sequences that contain the putative binding sites of miR-765 in GenePharma company (Shanghai, China). HEK293 cells were co-transfected with 20 mmol/L miR-765 mimic or miR-NC together with WT-LINC-FAM138B/Mut-LINC-FAM138B. Luciferase activity was measured with Dual Luciferase Reporter Assay Kit (Transgene, China) on GloMax20/20 at 48 h after the transfection.

### RIP

We used RIP assay to determine the binding between LINC-FAM138B and miR-765 using Magna RIP™ RNA-Binding Protein Immunoprecipitation Kit (Millipore) as previous study [34]. Briefly, HepG2 cells were transfected with biotinylated miR-765/miR-NC, and the mRNA level of linc-FAM138B or miR-765 was detected using qRT-PCR.

### Statistical analysis

Data were shown as mean±SD. Student’s t-test or one-way ANOVA was used to compare the groups. P<0.05 was considered significance. All the experiments were repeated in triplicate.

## Supplementary Material

Supplementary Figure 1
