# Supplementary material for: Exosomal linc-FAM138B from cancer cells alleviates hepatocellular carcinoma progression via regulating miR-765
Source: Aging (Albany NY). 2020 Dec 26;12(24):26236–47. doi: 10.18632/aging.202430 (PMC7803541; doi:10.18632/aging.202430)
Supplement: Supplementary Figure 1 [file aging-12-202430-s001.pdf]

SUPPLEMENTARY FIGURES

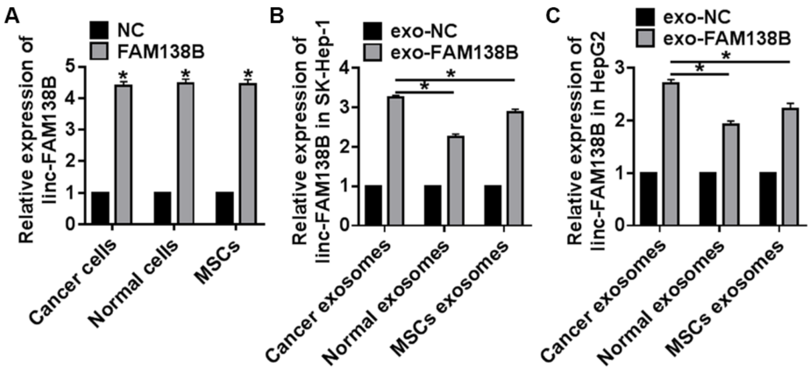

Supplementary Figure 1. Cancer cells transmitted exo-FAM138B into hepatocellular carcinoma cell lines. (**A**) Linc-FAM138B or NC was transfected into cancer cells, normal cells and MSCs, the expression of linc-FAM138B was tested by qRT-PCR. Exosomes were isolated from the supernatant of cancer cells, normal cells and MSCs after 36 h of transfection, then SK-Hep-1 (**B**) and HepG2 (**C**) cells were incubated with exosomes.
